# A Six-Degree-of-Freedom (6-DOF) Simultaneous Measurement Method Using Dual-Wavelength Laser Sources for Compensation of Air-Turbulence-Induced Beam Deviation

**DOI:** 10.3390/s25237122

**Published:** 2025-11-21

**Authors:** Fei Long, Xing Xia, Bin Zhang, Qibo Feng

**Affiliations:** Key Lab of Luminescence and Optical Information, School of Physical Science and Engineering, Beijing Jiaotong University, Beijing 100044, China

**Keywords:** 6-DOF measurement, air turbulence, dual-wavelength compensation, laser measurement

## Abstract

**Highlights:**

**What are the main findings?**

- We proposed a 6-DOF geometric error measurement method combining interferometry, collimation/autocollimation, and polarization.
- We developed a dual-wavelength proportional cancellation model to compensate for air turbulence.

**What are the implication of the main findings?**

- The method enables fast and accurate 6-DOF error measurement of linear guides with greatly improved efficiency over traditional single-parameter instruments.
- It enhances straightness accuracy in long-distance measurements by effectively suppressing air turbulence effects.

**Abstract:**

Linear guides are fundamental components of high-end precision equipment, and their geometric errors directly affect the measurement and machining accuracy. To achieve efficient and accurate measurement of geometric motion errors in linear guides, this paper proposes a 6-DOF simultaneous measurement method that integrates heterodyne interferometry, collimation/autocollimation, and polarization principles. To address the degradation of straightness measurement accuracy under long-distance conditions caused by air turbulence, a dual-wavelength laser-based compensation method is developed to suppress turbulence-induced beam deviation. A turbulence compensation model based on a dual-wavelength proportional cancellation principle is established, and its effectiveness is verified through COMSOL (v6.3) simulations and experimental studies. Experimental results show that the proposed approach significantly outperforms the traditional simple moving-average (SMA) filter. It improves straightness measurement stability by more than 56%. Under a 3200 mm measurement range and ordinary laboratory conditions, the repeatabilities (k = 2) of the 6-DOF motion-error measurements are 6.4 μm for positioning error, 6.4 μm and 5.5 μm for straightness errors, 1.7″ and 2.1″ for yaw and pitch errors, and 4.3″ for roll error. The proposed method exhibits high measurement accuracy and robustness, making it suitable for simultaneous 6-DOF motion-error measurement of long linear guides.

## 1. Introduction

Accurate measurement of geometric errors in linear guides is fundamental to achieving high-precision machining of machine tools [1]. During motion, a linear guide inevitably exhibits 6-DOF geometric errors, including translational errors along the X, Y, and Z axes and rotational errors about these axes. Traditional geometric-error measurement instruments such as micrometers, gauge blocks, precision levels, autocollimators, and laser interferometers [2,3,4] provide high accuracy and are easy to operate. However, they usually require separate measurement of each error component, which results in complicated procedures and low efficiency and cannot meet the growing demand for fast, high-efficiency measurements in modern manufacturing.

To achieve efficient and precise error measurement, multi-DOF measurement methods based on laser interferometry, collimation, and autocollimation principles have attracted increasing research attention because of their simple configuration and relatively low cost. Lee et al. [5] used gratings and a corner-cube reflector (RR) as error-sensitive elements and achieved 6-DOF error measurement through complex positional reconstruction. Shimizu et al. and Kimura et al. [6,7] introduced a Michelson interferometric configuration into a grating system to develop a 6-DOF grating encoder. Hsieh et al. [8] extended several interferometric techniques on a two-dimensional grating and realized simultaneous 6-DOF error measurement with displacement and angular resolutions of 2 nm and 0.01″, respectively. Cui [9] proposed a quasi-common-path heterodyne grating interferometric system that improved anti-interference capability. Jia [10] developed a 6-DOF measurement system using a polarization-maintaining-fiber-coupled dual-frequency laser and an RR as the error-sensitive element, achieving a positioning-error resolution of 1 nm, straightness resolution of 0.1 μm, pitch and yaw resolutions of 0.26″, and roll-angle resolution of 0.69″. Zhou [11] constructed a dual-comb three-DOF measurement system combining gratings and RR, obtaining a positioning accuracy of 12.7 nm and angular accuracy of 0.088″. Yu [12] proposed a 6-DOF measurement system based on differential wavefront sensing and polarization detection.

Overall, these multi-DOF measurement methods offer large measurement ranges, low cost, and high integration, demonstrating significant application potential. However, maintaining long-term accuracy and stability under environmental disturbances remains a major challenge [13,14].

The collimation method is one of the most commonly used techniques for straightness measurement and is also frequently employed in 6-DOF error-measurement systems. In long-distance measurements, the stability of laser propagation in air directly determines measurement accuracy. Fluctuations in environmental parameters such as temperature, pressure, and humidity generate airflow vortices of different scales, which interact to form random air turbulence [15]. Turbulence along the propagation path severely degrades beam stability and causes beam wander, becoming one of the principal factors that limit the precision and stability of long-range straightness measurements [16].

To suppress the straightness instability caused by air turbulence, several approaches have been developed, including common-path compensation, feedback control, environmental isolation, and signal-filtering methods. Feng [17] proposed a common-path digital compensation method to suppress beam drift. Huang [18] employed mirror-control technology to monitor and correct laser drift in real time. Liu [19] designed a turbulence-shielding cover, and Li [20] utilized bellows to enhance system stability. Wang [21] analyzed turbulence characteristics and developed an ARMA algorithm to mitigate the effects of air disturbance. However, some of these techniques, such as physical shielding, are difficult to implement in practice. ARMA is essentially a time-domain smoothing technique. When turbulence-induced disturbances overlap in frequency with the true motion or are non-stationary, it is difficult to separate the two without distortion; increasing the filter strength typically introduces amplitude attenuation and phase delay, thereby degrading the fidelity of the true displacement.

To suppress turbulence while preserving the real displacement, we propose a dual-wavelength proportional cancellation method. Leveraging air dispersion, the turbulence-induced deflection amplitudes at two wavelengths that propagate along the same path exhibit a near-proportional relationship. This allows the common-mode disturbance to be canceled, suppressing turbulence while retaining the true geometric displacement. Building on this, we integrate heterodyne interferometry, collimation/autocollimation, and polarization-based sensing to develop a 6-DOF simultaneous measurement system with turbulence compensation. Experiments validate the effectiveness and accuracy of both the measurement scheme and the compensation method.

## 2. Methodology

**Figure 1:**
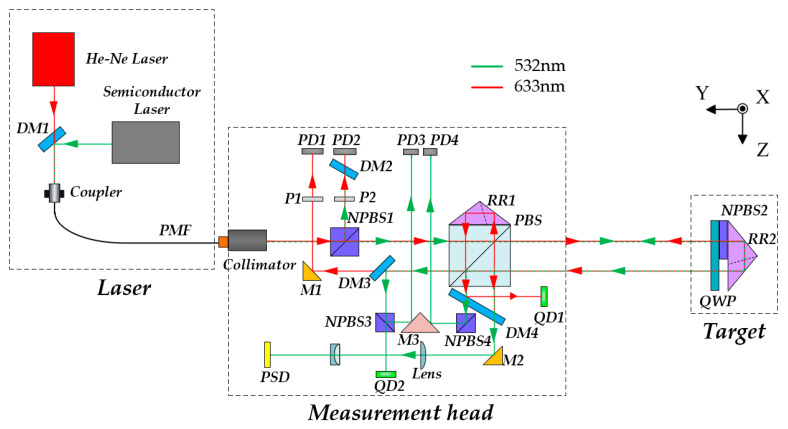
Schematic diagram of the proposed 6-DOF measurement method.

The proposed 6-DOF measurement method is illustrated in Figure 1. It consists of three main parts: the laser unit, the measurement unit, and the target unit. The laser unit includes a dual-frequency He-Ne laser (λ = 632.8 nm), a semiconductor laser (λ = 532 nm), and the corresponding coupling components. The two laser beams are combined by dichroic mirror 1 (DM1, Longpass dichroic mirror, 605 nm cut-on wavelength) and then coupled into a polarization-maintaining fiber (PMF, ≥18 dB) to stabilize the input polarization state and reduce thermal effects in the system. The two laser beams are transmitted through the PMF to the measurement unit, where they are collimated into parallel beams by an achromatic collimator. The He-Ne laser is mainly used for heterodyne-interferometric measurement of positioning errors and for providing a reference beam for laser-drift compensation, while the semiconductor laser is employed for measuring the other five degrees of freedom.

After collimation, the He-Ne laser beam first passes through non-polarizing beamsplitter cubes 1 (NPBS1) for beam splitting. The reflected beam passes through a high-extinction polarizer P2 (Tp:Ts > 5000:1) to set a well-defined polarization state, after which it generates the reference heterodyne signal at photodiode PD2. A dichroic mirror (DM2) is placed in front of PD2 to suppress any residual 532 nm light and avoid unintended interference. The beam transmitted through NPBS1 is directed to a polarizing beam splitter (PBS, Tp:Ts > 1000:1), which separates it into two beams according to their optical frequencies (*f*_1_ and *f*_2_). The reference beam (*f*_1_) is reflected by a fixed RR1 and then successively reflected by PBS and mirror 1 (M1) toward PD1. The measurement beam (*f*_2_) exits the measurement unit, passes twice through a quarter-wave plate (QWP, ±λ/300) whose fast axis is oriented at 22.5° relative to the *y*-axis, thereby forming a circularly polarized beam. This beam is retroreflected by the movable RR2. When passing through PBS again, the beam is split into two paths: one transmitted beam is reflected by M1 and interferes with the reference beam to generate the beat signal, while the other reflected beam is directed to quadrant photodiode 1 (QD1) via DM4 to provide reference compensation for beam-drift suppression.

After being transmitted through the PBS, the semiconductor laser beam enters the target unit, where it is divided into two paths. One of the beams is reflected by NPBS2 and passes twice through QWP for polarization modulation. When it returns to the PBS, the s-polarized component is reflected and directed by M2 through the focusing lens group (*f* = 250 mm), where it is finally focused onto the position-sensitive detector (PSD, resolution 0.7 μm) to measure the pitch and yaw angles. The other transmitted beam passes through NPBS2 and is reflected by an RR. The retroreflected beam carries two-dimensional straightness and roll-angle information. It is split by PBS, with the reflected beam directed to PD4 via M3 and NPBS4, and the transmitted beam reflected to PD3 via DM3, NPBS3, and M3. The differential output between PD3 and PD4 yields the roll angle error. The beam transmitted by NPBS3 is incident on QD2 for straightness measurement, which is compensated in real time by the reference signal from QD1.

### 2.1. Principle of Measurement System

#### 2.1.1. Principle of Positioning Error Measurement

**Figure 2:**
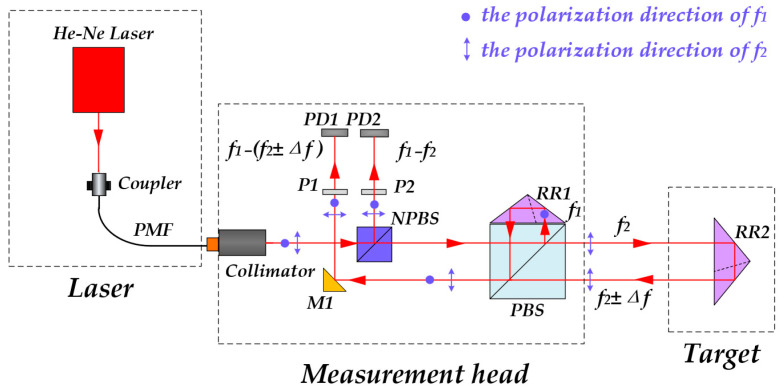
Schematic diagram of the interferometric measurement principle.

The positioning error is measured using a heterodyne interferometric method, as illustrated in Figure 2. The dual-frequency He–Ne laser emits two orthogonally linearly polarized beams with frequencies *f*_1_ and *f*_2_. When coupled into the polarization-maintaining fiber, the polarization directions of the two beams are aligned with the fast and slow axes of the fiber, respectively, ensuring polarization stability and minimizing polarization crosstalk during transmission [22]. After transmission through the polarization-maintaining fiber, the dual-frequency beams enter the measurement unit and are first divided into two paths by the NPBS. One reflected beam is directed to PD2 through P2 to form the reference beat-frequency signal with a frequency given by:

(1) $$\Delta f_{r}=f_{1}-f_{2}=\Delta f_{0}$$

When the transmitted beam passes through the PBS, the collimator’s installation angle can be finely adjusted so that the orthogonally polarized beams are precisely separated by the PBS into a transmitted beam (*f*_2_) and a reflected beam (*f*_1_). The presence or absence of a beat frequency in the transmitted beam can be observed to determine whether the laser frequency is spectrally pure. The *f*_1_ component serves as the reference beam and is reflected by RR1, M1, and P1 before being detected by PD1. The *f*_2_ component acts as the measurement beam, passing through the PBS and reflected by RR2, M1, and P1 before being detected by PD2. The interference between the reference and measurement beams generates a heterodyne signal at PD2, whose frequency is expressed as:

(2) $$\Delta f_{m}=f_{1}-(f_{2}\pm \Delta f)=\Delta f_{0}\pm \Delta f$$

Here, ±Δ*f* represents the Doppler frequency shift caused by the motion of the retroreflector platform, where Δ*f* = (2*π* × 2*v*)/*λ* and *v* denotes the moving velocity of RR2.

According to the relationship between frequency and phase

(3) $$\begin{array}{c} \Delta f=\frac{d(\Delta \varphi )}{dt}\\ \Delta \varphi =\int_{0}^{t}\Delta fdt=\int_{0}^{t}\frac{2\pi \times 2\upsilon }{\lambda }dt=\frac{4\pi }{\lambda }\Delta L \end{array}$$

where Δ*φ* represents the phase difference between the measurement and reference signals, and Δ*L* denotes the displacement of the moving platform.

#### 2.1.2. Principle of Straightness Measurement

**Figure 3:**
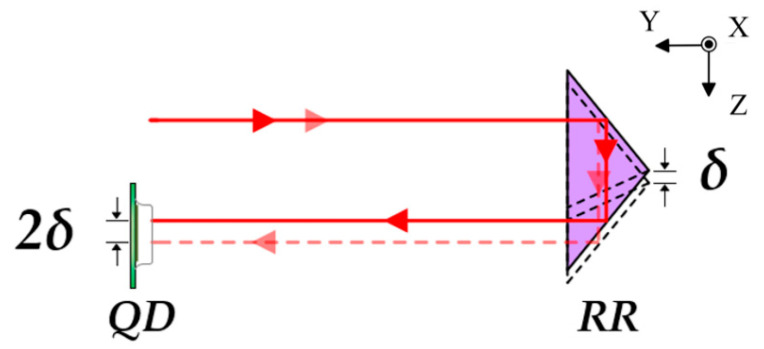
Principle of straightness measurement based on laser collimation.

**Figure 4:**
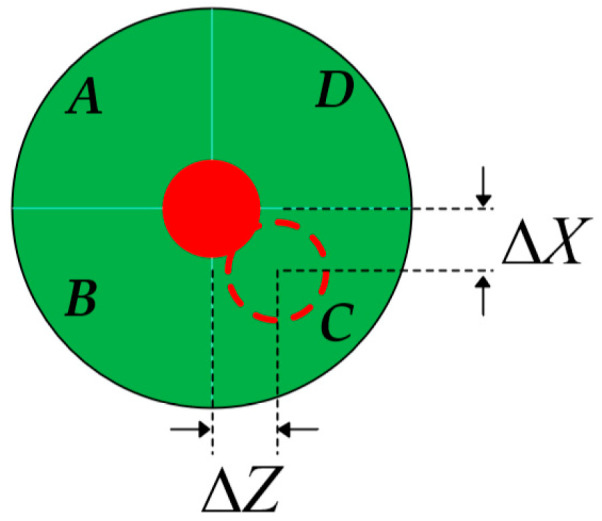
Schematic of QD.

The principle of straightness measurement based on laser collimation is illustrated in Figure 3. An RR is employed as the straightness-sensitive element. When the prism is displaced, the position change of the outgoing beam doubles that of the prism itself, thereby achieving enhanced measurement sensitivity. The QD consists of four photodiodes with identical performance, as shown in Figure 4. When the laser beam deviates from the center of the detector, the displacement of the beam spot can be expressed as

(4) $$\Delta Y_{QD}=k\frac{I_{A}+I_{D}-\left(I_{B}+I_{C}\right)}{I_{A}+I_{B}+I_{C}+I_{D}},$$

(5) $$\Delta X_{QD}=k\frac{I_{A}+I_{B}-\left(I_{C}+I_{D}\right)}{I_{A}+I_{B}+I_{C}+I_{D}},$$

where *I_A_*, *I_B_*, *I_C_*, and *I_D_* represent the optical power received by the four quadrants of the detector, and *k* is the conversion coefficient.

#### 2.1.3. Principle of Pitch and Yaw Angle Measurement

**Figure 5:**
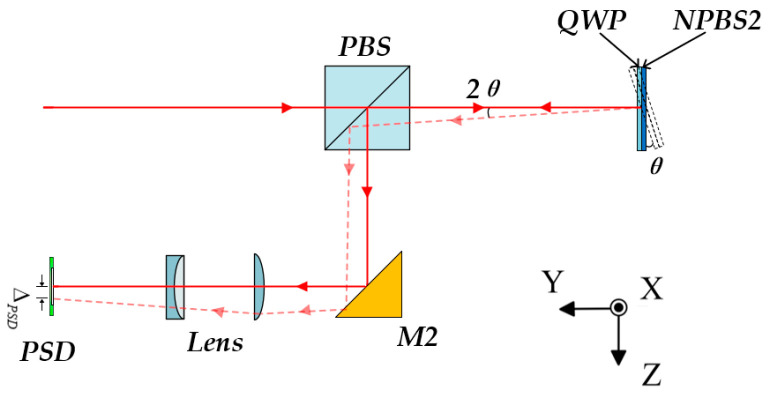
Principle of pitch and yaw angle measurement based on autocollimation.

As illustrated in Figure 5, the NPBS2 serves as a two-dimensional angle-sensitive element (the solid line represents the optical path without angular deviation, while the dashed line indicates the optical path when an angular deviation is present). According to the principle of autocollimation, when NPBS2 undergoes a small angular deflection of *θ*, the reflected laser beam experiences an angular variation of *2θ*. The deflected beam is focused onto the PSD by a lens group, and the displacement of the light spot output from the PSD can be used to calculate the corresponding angular change. The relationship between the angular variation *θ* and the focal length *f* of the lens is expressed as:

(6) $$\tan2\theta =\frac{\Delta_{PSD}}{f},$$

When *θ* is very small, it can be approximated as:

(7) $$\theta =\frac{\Delta_{PSD}}{2f}$$

#### 2.1.4. Principle of Roll Angle Measurement

**Figure 6:**
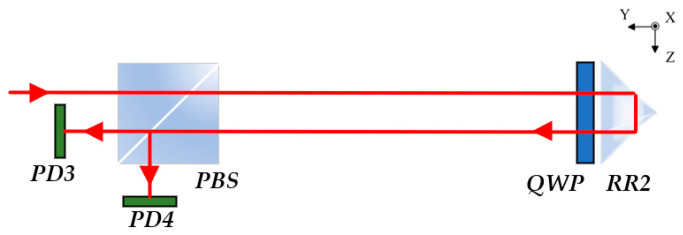
Principle of roll angle measurement based on laser polarization.

The measurement of roll angle is based on the laser polarization principle, as illustrated in Figure 6. A QWP is employed as the roll-angle-sensitive element. When the RR rotates slightly about the *z*-axis, the light intensities received by the two PDs change accordingly. By performing differential processing of the output voltages from the two detectors, both the magnitude and direction of the roll angle can be obtained.

Let *I*_0_ denote the emitted laser intensity. When the optical axis of the QWP forms an angle of 22.5° + *γ* with the laser polarization direction, the relationship between the intensity difference of the two detectors and the roll angle *γ* can be expressed as [23]:

(8) $$\Delta I=-I_{0}\sin(4\gamma )=-\frac{I_{0}\pi }{180}[4\gamma +ο(\gamma^{3})]$$

### 2.2. Principle of Air-Turbulence Compensation

Due to the dispersion effect of air, the response amplitudes of refractive-index variations differ proportionally for different wavelengths [24]. Two laser beams of different wavelengths propagating along the same optical path experience nearly identical but not completely correlated refractive-index fluctuations in the same turbulent field. By utilizing the dispersion ratio between the two wavelengths, the common turbulent components can be effectively estimated and eliminated, thereby improving the discrimination of the true geometric quantities.

**Figure 7:**
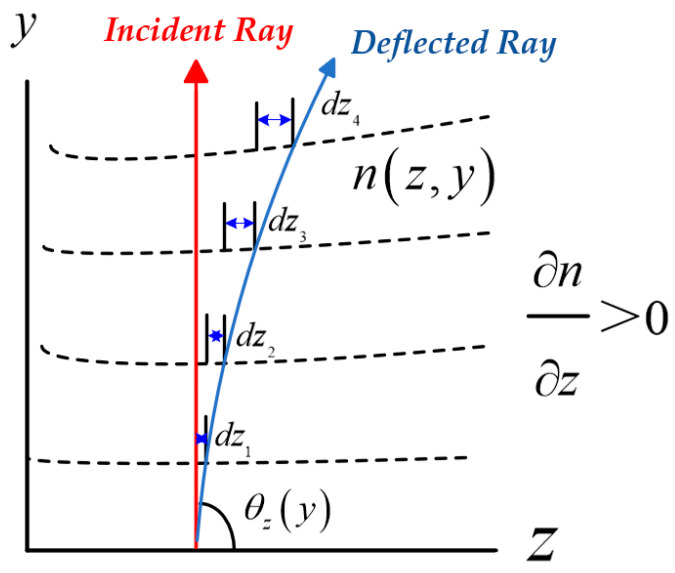
Schematic diagram of beam deviation caused by air turbulence.

As illustrated in Figure 7, the propagation direction of the beam without turbulence is assumed to be along the *y*-axis, while the deflection angle *θ_z_* represents the deviation of the beam in the *z*-axis under actual turbulent conditions. Assuming that the refractive-index distribution is *n*(*z*,*y*,*λ*), and under the small-angle and weakly inhomogeneous medium approximations, the relationship can be described by the Eikonal equation [25]:

(9) $$\frac{d}{dy}\theta_{z}\approx \frac{1}{n_{0}\left(\lambda \right)}\frac{\partial n}{\partial z},$$

where *n*_0_ represents the background refractive index. The lateral displacement of the beam spot at the detector (QD) can be expressed in integral form as:

(10) $$\Delta z(y,\lambda )=d_{z_{1}}+d_{z_{2}}+\cdot \cdot \cdot d_{z_{n}}=\frac{1}{n_{0}\left(\lambda \right)}\int_{0}^{y}\int_{0}^{y^{\prime }}\frac{\partial n(z,y^{\prime \prime })}{\partial z}dy^{\prime \prime }dy^{\prime }$$

Equation (10) indicates that the lateral displacement of the beam spot is determined by the integral of the transverse refractive-index gradient along the propagation path.

Based on Ciddor’s air refractive-index dispersion model [26], the refractive index can be expressed as the sum of a background term and a fluctuation term:

(11) $$n\left(z,y,\lambda \right)=n_{0}\left(\lambda \right)+\delta n(z,y)\beta \left(\lambda \right),$$

where *δn*(*x,z*) represents the spatial fluctuation amplitude caused by temperature or density variations, and *β*(*λ*) is the wavelength-dependent dispersion coefficient.

Let the two wavelengths be denoted as *λ*_1_ and *λ*_2_, and define the deflection amplitude ratio *a* between the two wavelengths as:

(12) $$a=\frac{\Delta z\left(\lambda_{1}\right)}{\Delta z\left(\lambda_{2}\right)}$$

Combining Equations (10) and (11), we obtain:

(13) $$a\approx \frac{\beta \left(\lambda_{1}\right)/n_{0}\left(\lambda_{1}\right)}{\beta \left(\lambda_{2}\right)/n_{0}\left(\lambda_{2}\right)}\approx \frac{n\left(\lambda_{1}\right)-1}{n\left(\lambda_{2}\right)-1}$$

When *λ*_1_ = 532 nm and *λ*_2_ = 632.8 nm, *a* = 1.006.

Let the displacement signals measured by the detectors be *S*_1_(*t*) and *S*_2_(*t*). Considering the double-pass amplification of the RR and the calibration factor, their relationship with the true geometric quantity *δ_true_*(*t*) can be expressed as:

(14) $$\left\{\begin{array}{c} S_{1}(t)=\delta_{true}(t)+\frac{1}{2}\Delta_{1}(\lambda_{1},t)+{ο}_{1}\left(t\right)\\ S_{2}(t)=\delta_{true}(t)+\frac{1}{2}\Delta_{2}(\lambda_{2},t)+{ο}_{2}\left(t\right)\\ \Delta_{1}(t)=a\Delta_{2}(t) \end{array}\right.,$$

where Δ_*i*_(*λ_i_*,*t*) denotes the beam deflection induced by air turbulence, and *o_i_*(*t*) represents the detector’s self-measurement noise. When the two detection channels are noise-free and perfectly matched, the true measured displacement can be obtained through proportional elimination as:

(15) $$\delta_{true}\left(t\right)=\frac{S_{1}(t)-aS_{2}(t)}{1-a}$$

In practice, since 1 − *a* ≪ 1, direct use of the proportional-cancellation equation would lead to excessive amplification of detector noise. Therefore, frequency-domain analysis and filtering are required in the compensation process, which can be divided into three main steps:

1. Preprocessing

In addition to turbulence-induced fluctuations, the original straightness measurement data may contain occasional spikes such as sampling dropouts or detector saturation, which can produce abnormal energy in the power spectral analysis. Therefore, a sliding median filter with a window size of 5 is first applied to the data, which effectively suppresses impulsive noise while keeping the subsequent power spectrum and coherence analyses unaffected.

2. Selection of the Processing Bandwidth

In indoor environments (within approximately 10 m), the spatial spectrum of turbulence approximately follows the Kolmogorov–Obukhov scaling law (κ − 5/3). Under the Taylor frozen-flow hypothesis, the temporal frequency can be mapped from the equivalent mean flow velocity *U* as [27]:

(16) f≈U/l,

where *U* = 0.2–1 m/s is the mean flow velocity, and *l* = 0.1–0.5 m is the effective eddy size. Accordingly, the dominant turbulence energy corresponds to the frequency range of approximately 0.01–10 Hz. To avoid mechanical drift and electronic noise from the detection circuit, the final processing bandwidth is limited to 0.05–3 Hz. This range covers most of the turbulence energy while excluding ultra-low-frequency drift and high-frequency noise [28]. Direct elimination across the entire 0.05–3 Hz band would also remove components unrelated to turbulence; hence, a coherence threshold is introduced for selective frequency-domain compensation to prevent noise amplification.

**Figure 8:**
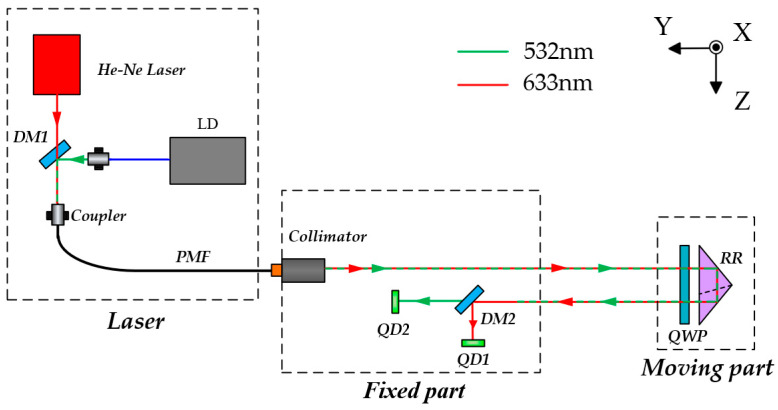
Principle of dual-wavelength proportional-cancellation model for air-turbulence compensation.

Figure 8 shows the optical configuration of the dual-wavelength straightness measurement system. The two laser beams share an identical propagation path, so the refractive-index fluctuations induced by turbulence exhibit highly correlated spectral characteristics. The Welch method is used to estimate the power spectral densities *S*_11_(*f*) and *S*_22_(*f*) of the two signals, as well as their cross-spectral density *S*_12_(*f*). The coherence coefficient is then calculated as [29]:

(17) $$\gamma^{2}\left(f\right)=\frac{{\left|S_{12}(f)\right|}^{2}}{S_{11}\left(f\right)S_{22}\left(f\right)},$$

When *γ*^2^ ≥ 0.8^2^, the two signals are considered strongly coherent, and the frequency components in this region are retained for effective proportional cancellation and turbulence compensation.

3. Signal Compensation

Under the ideal dispersion condition, the disturbances at the two wavelengths satisfy a strict proportional relationship. However, the actual system inevitably involves various errors, such as circuit gain mismatch and slight differences in the beam sizes of the two optical paths, which may cause small deviations in the proportional coefficient used for cancellation. Therefore, $\hat{a}$ is estimated by least squares by minimizing the residual of *S*_1_ − *aS*_2_. The expression for $\hat{a}$ is:

(18) $$\hat{a}=\frac{C\mathrm{ov}\left(S_{1},S_{2}\right)}{Var\left(S_{2}\right)}$$

According to the Wiener–Khinchin theorem, the covariance and variance can be written as spectral integrals:

(19) $$\begin{array}{l} Cov\left(S_{1},S_{2}\right)=\int_{-\infty }^{\infty }S_{12}\left(f\right)df\\ Var\left(S_{2}\right)=\int_{-\infty }^{\infty }S_{22}\left(f\right)df \end{array}$$

Hence,

(20) $$\hat{a}=\frac{\int S_{12}\left(f\right)df}{\int S_{22}\left(f\right)df}$$

Following Equation (17), proportional cancellation is performed only in the high-coherence band. Therefore,

(21) a^f=ReS12fS22f,f∈High coherence

Based on this, the frequency-domain cancellation operator is constructed as:

(22) $$H\left(f\right)=\frac{1}{1-\;\hat{a}\left(f\right)+\varepsilon },$$

where *ε* = 10^−2^ is a regularization term introduced to prevent excessive amplification of noise when $\hat{a}$(*f*)→1. To adaptively control the compensation strength according to the signal coherence level, a weighting function is defined as:

(23) $$W\left(f\right)=\gamma^{2}{\left(f\right)}^{p},\;p\in \left[1,1.5\right],$$

where *p* can be either a function of the coherence threshold or a fixed constant. In this work, we set *p* = 1.2 empirically.

Accordingly, the compensated spectrum can be obtained as:

(24) $$\hat{\delta }\left(f\right)=W\left(f\right)H\left(f\right)\left[X_{1}\left(f\right)-\hat{a}\left(f\right)X_{2}\left(f\right)\right]+\left(1-W\left(f\right)\right)X_{1}\left(f\right),$$

where *X*_1_(*f*) and *X*_2_(*f*) denote the Fourier spectra (frequency-domain representations) of *S*_1_(*t*) and *S*_2_(*t*), respectively.

Finally, the time-domain compensated signal sequence *δ*(*t*) is reconstructed via the inverse fast Fourier transform (IFFT).

## 3. Simulation Verification

To verify the effectiveness of the dual-wavelength proportional-cancellation model in Equation (15), a numerical simulation coupling ray tracing and fluid dynamics was established using Comsol Multiphysics. The model incorporated three modules: fluid dynamics, heat transfer, and geometric optics configuration, which together simulated the effect of air turbulence on laser-beam propagation.

**Figure 9:**
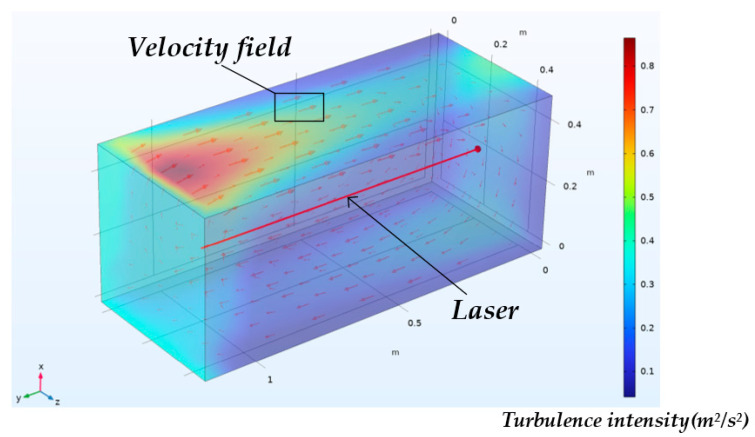
Numerical model in Comsol Multiphysics coupling ray tracing and fluid dynamics.

As shown in Figure 9, the domain is a rectangular sealed cavity of 1.2 × 0.6 × 0.6 m. A velocity inlet is applied at the inlet with flow directed along −y and a small periodic perturbation superposed to emulate turbulence; the outlet is a static-pressure outlet (0 Pa). All solid walls use the no-slip condition, and the two side faces are set as symmetry planes. To induce natural convection and turbulence, a constant heat flux of 2 W/m^2^ is imposed on one wall, while the opposite wall is maintained at 293.15 K, creating a stable temperature gradient. The light source is placed at the inlet plane, and rays propagate along −y, coupled to the velocity and refractive-index fields to compute beam deflection. Full boundary conditions and parameters are provided in Appendix A.

During turbulence, the beam spot position was sampled over a total duration of 0.1 s with a sampling interval of 0.01 s, recording the time-varying positions at the two wavelengths (633 nm and 532 nm).

**Figure 10:**
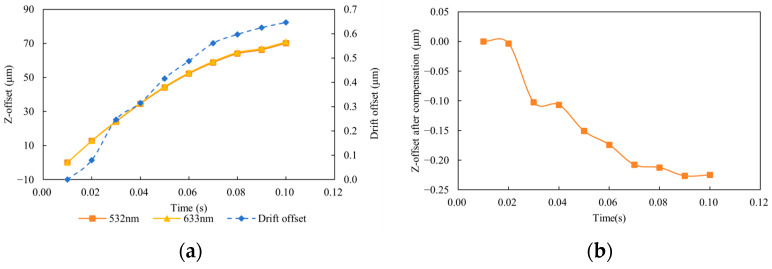
Simulation results. (**a**) Z-offsets at 532 nm and 633 nm and their drift offset; (**b**) Z-offset after compensation.

As shown in Figure 10, taking the beam position variation along the z-direction as an example, the two beams exhibit a displacement of approximately 70 μm within 0.1 s, and the difference between the two wavelengths is 0.65 μm. According to Equations (13) and (15), the compensated residual does not exceed 0.25 μm. The error was reduced by 99.6%. However, a small residual error remains, which may be attributed to the approximations in the compensation model. When the turbulence intensity becomes stronger, the proportional relationship between the two wavelengths weakens, resulting in reduced compensation effectiveness. Therefore, neglecting electronic noise, the dual-beam turbulence compensation effectively suppresses turbulence-induced beam wander, demonstrating the validity of the dual-wavelength proportional cancellation model.

## 4. Experiment and Analysis

Based on the proposed 6-DOF simultaneous measurement method and the air-turbulence compensation principle, a high-precision measurement system was constructed for the simultaneous detection of six geometric error components. A series of experiments was conducted to verify the feasibility and effectiveness of the proposed method. All experiments were performed in an open laboratory without environmental control. The standard reference instruments used in the experiments included: a linear encoder (ESSA Technology Co., Ltd., Qingdao, China; model: LG-50; measurement accuracy: 0.1 μm; resolution: 0.05 μm); an autocollimator (Angco Optoelectronics, Xi’an, China; measurement accuracy: 0.15 arcsec; resolution: 0.01 arcsec); a laser interferometer (Renishaw, Gloucestershire, UK; model: XL-80; linear measurement accuracy: ±0.5 ppm; angular accuracy: ±(0.002A + 0.5 + 0.1M) µrad; straightness measurement accuracy: ±(0.005A + 0.5 + 0.15M) µm); and an electronic level (Qianxiao Electronics, Qingdao, China; resolution: 0.001 mm/m).

### 4.1. Calibration Experiment

To verify the performance of the proposed measurement system, a series of calibration experiments was conducted in the laboratory. The environmental conditions during the experiments were as follows: air temperature ranged from 20 ± 0.2 °C, relative humidity was 20.5 ± 1%, and atmospheric pressure was 1.01 k mbar.

The LG-50 linear encoder was employed to calibrate the straightness measurement error, while an autocollimator was used to calibrate the angular measurements, including pitch and yaw angles as well as roll angle. The calibration ranges were ±100 μm for displacement and ±100″ for angular measurements, with calibration intervals of 20 μm and 20″, respectively. The results show that, compared with the standard instruments, the maximum relative deviation in straightness measurement was 0.4 μm, and the maximum relative deviation in angular measurement was 0.5″.

### 4.2. Air-Turbulence Compensation Experiment

**Figure 11:**
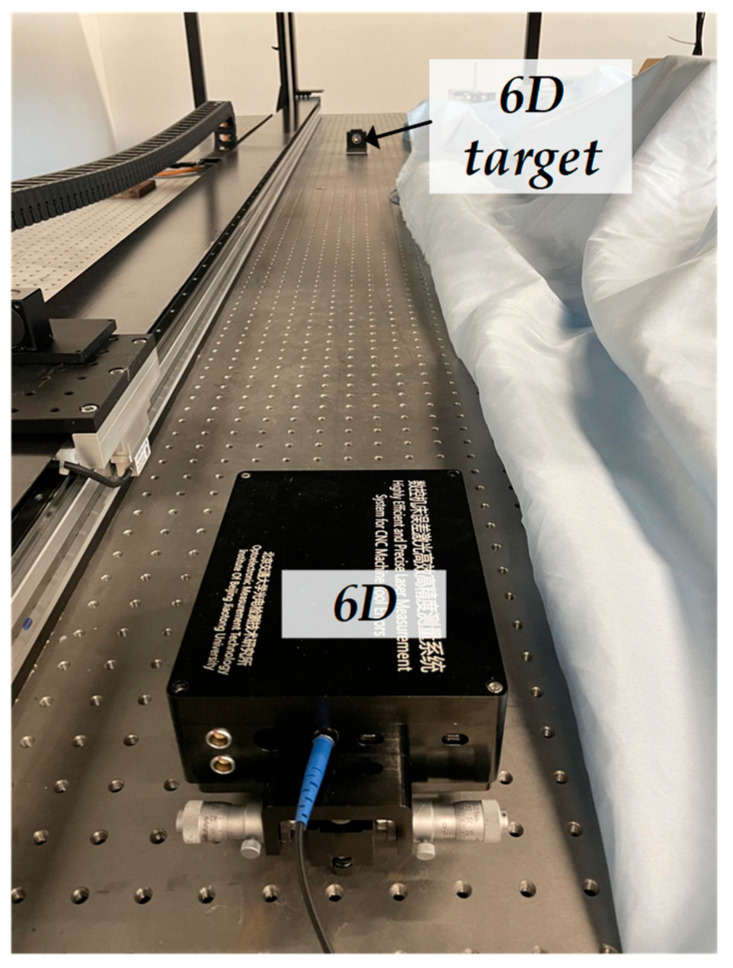
Experimental setup for straightness stability test.

**Figure 12:**
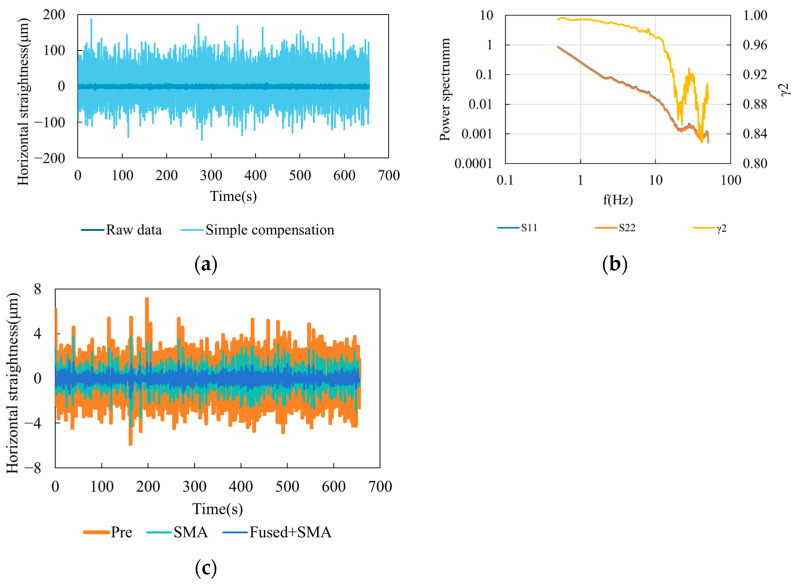
Horizontal straightness stability results. (**a**) Comparison of raw data and simple compensation results. (**b**) Power spectral density and coherence of the dual-wavelength signals; (**c**) Horizontal straightness stability results before and after compensation.

**Figure 13:**
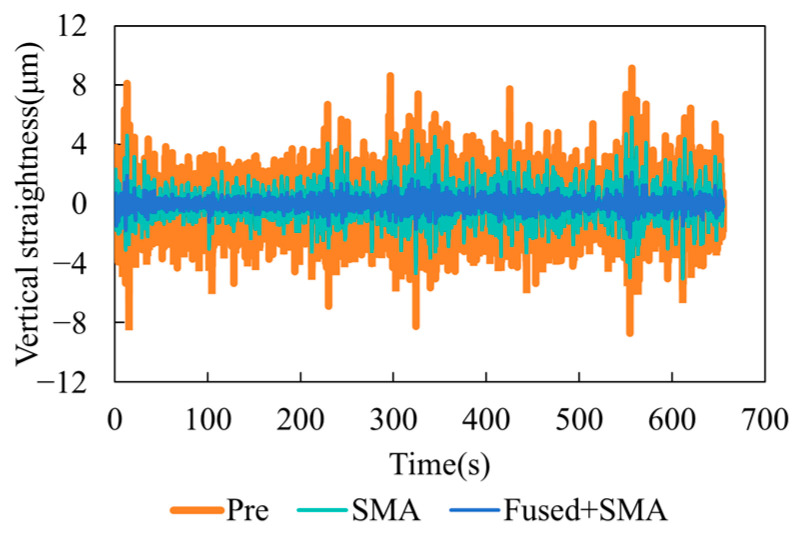
Vertical straightness stability results.

To verify the effectiveness of the air-turbulence compensation method, a straightness stability experiment was conducted using the 6-DOF measurement system at a working distance of 2 m. As shown in Figure 11, both the fixed unit and the target unit were mounted on an optical platform. The data acquisition frequency was 100 Hz, and the entire experiment lasted for ten minutes. The experimental results are presented in Figure 12 and Figure 13.

Figure 12a compares the raw data with the results obtained using the simple cancellation model described in Equation (15). It can be observed that when the simple cancellation model is directly applied to the entire dataset, the small denominator in the equation greatly amplifies the noise contained in the detector outputs, resulting in severe signal distortion. Therefore, frequency-domain processing and analysis are required to mitigate the influence of noise and ensure reliable compensation performance.

Figure 12b shows the power spectral density and coherence spectra of the two laser wavelengths. n the main frequency band of 0.1–3 Hz, the two spectra almost overlap and exhibit a red-noise-like characteristic, indicating resonance behavior within the common turbulent optical path. In the higher-frequency region, the spectral energy decreases and the two signals diverge due to sensor and electronic noise. The coherence coefficient *γ^2^* remains within the range of 0.8–0.95 across 0.25–2.75 Hz. Therefore, the entire frequency band meets the compensation requirement. Figure 12c presents the horizontal straightness data, including the original signal (Pre, orange), the SMA-filtered result (green), and the dual-wavelength compensated result (Fused + SMA, blue). The original signal exhibited random fluctuations with a peak-to-peak value of 12.9 μm. After SMA filtering (smoothing window of 50 data points, i.e., 0.5 s), the noise amplitude was reduced to 8.1 μm but remained unstable. With dual-wavelength compensation, the peak-to-peak value was further reduced to 3.4 μm, accounting for only 42% of the filtered value. The vertical straightness results are shown in Figure 13, and a summary of the statistical results is given in Table 1. Compared with SMA filtering, the air-turbulence compensation reduced the standard deviation and peak-to-peak values of horizontal straightness by 56% and 58%, respectively, and those of vertical straightness by 67% and 60%, respectively. These results confirm the effectiveness of the proposed air-turbulence compensation method.

### 4.3. Guideway Measurement Comparison Experiment

**Figure 14:**
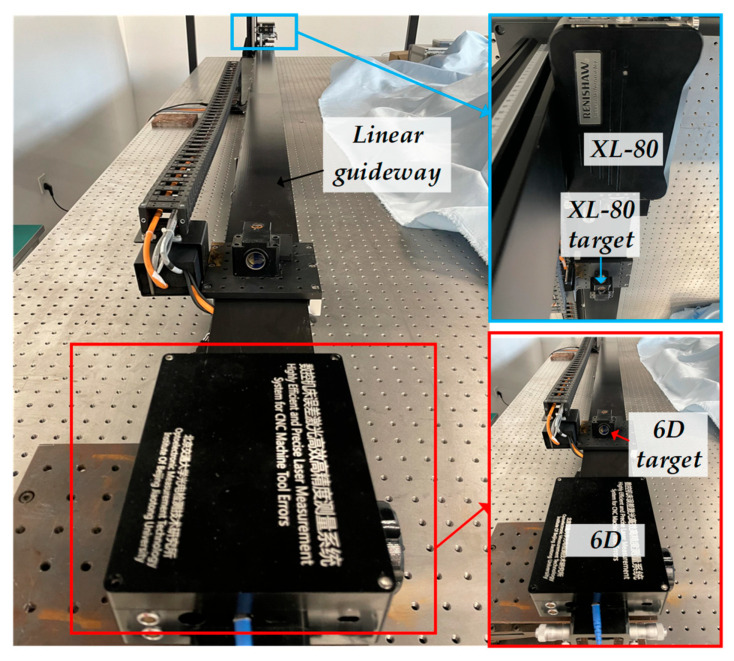
Photo of the repeatability experiment setup.

A commercial instrument, the RENISHAW XL-80 laser interferometer (Wotton-under-Edge, UK), was used as the reference standard to perform repeatability and comparative experiments for 6-DOF geometric error measurements. As shown in Figure 14, both the 6D laser measurement system and the XL-80 measurement head were mounted on the same optical platform, while the target unit was installed on a linear guideway. The height of the target unit was carefully adjusted to match that of the XL-80. The total measurement distance was set to 3200 mm, with an interval of 200 mm between adjacent measurement points. Each measurement point was held for 6 s, and the entire measurement process was repeated three times to evaluate repeatability. The total duration of the repeatability experiment was approximately 10 min.

**Figure 15:**
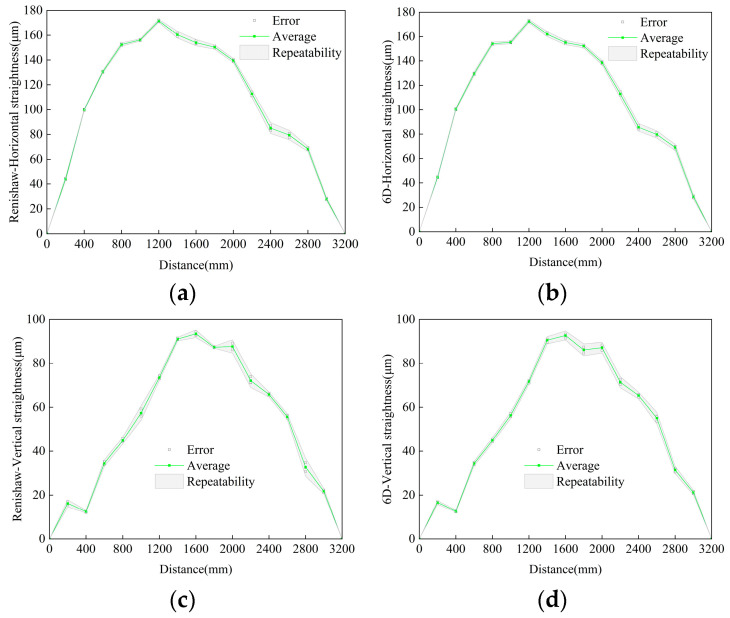
Straightness experiment results. (**a**) Renishaw horizontal straightness measurement result; (**b**) 6D horizontal straightness measurement result; (**c**) Renishaw vertical straightness measurement result; (**d**) 6D vertical straightness measurement result.

Figure 15 shows the mean values and repeatabilities measured by the two instruments. To verify the feasibility of the air-turbulence compensation method in practical straightness measurements, the data acquired during each 6 s dwell period at every measurement point were processed using both the SMA filtering and the dual-wavelength compensation methods. The median of each time sequence was selected as the measurement error value for that position. According to the ISO 230-2 standard [30], the repeatability R_i_ at a given position is calculated as R_i_ = 4S, corresponding to a 95% confidence level. The summarized straightness deviation results are listed in Table 2. After SMA filtering, the maximum deviations of straightness (within the 3200 mm measurement range, among all measured points) compared with the XL-80 reference were 3.6 μm and 3.0 μm, with repeatabilities of 9.8 μm and 9.3 μm. After applying the dual-wavelength compensation, the maximum deviations were reduced to 2.0 μm and 1.8 μm, with repeatabilities of 6.4 μm and 5.5 μm, respectively. The repeatabilities measured by the XL-80 itself were 8.8 μm and 8.2 μm. Compared with the traditional SMA filtering method, the dual-wavelength compensation reduced the maximum deviation by over 40% and improved the repeatability by more than 35%. Moreover, the repeatability of the dual-wavelength compensation method was superior to that of the Renishaw system. These results further demonstrate the effectiveness of the proposed dual-wavelength compensation method in practical straightness measurements.

**Figure 16:**
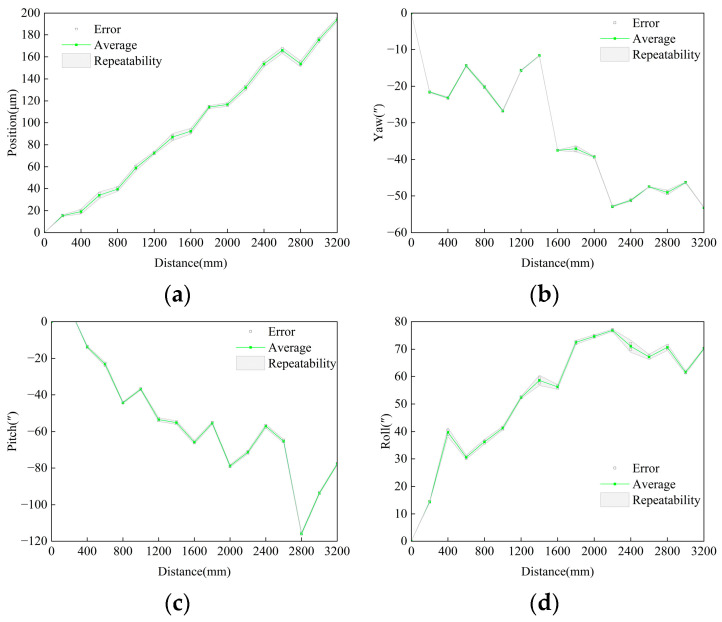
Other Degree-of-Freedom results of 6D. (**a**) Positioning; (**b**) Roll; (**c**) Yaw; (**d**) Pitch.

Figure 16 presents the measurement results of the other degrees of freedom of 6D, and the corresponding data are summarized in Table 3. The results show that, compared with the XL-80, the maximum deviation in positioning error measured by the 6D system was 2.5 μm, the maximum deviation in yaw angle was 4.1″, in pitch angle was 1.7″, and in roll angle was 4.6″. The angular measurement repeatability of the 6D system is generally consistent with that of the XL-80.

### 4.4. Result Analysis

The experimental results demonstrate that the air-turbulence compensation method outperforms the SMA filtering approach in both stability and repeatability tests. As shown in Figure 12 and Figure 13, the amplitude of signal fluctuations significantly decreased after compensation. As illustrated in Figure 15, the comparative and repeatability deviations of straightness errors were substantially reduced. These findings indicate that the proposed compensation method effectively eliminates random errors caused by refractive-index fluctuations within the dominant turbulence frequency band, thereby improving the anti-interference capability of long-distance measurements. However, residual noise remains in the compensated data, mainly due to out-of-band turbulence energy, turbulence anisotropy, imperfectly common optical paths, and uncertainty in the proportional estimate. In the future, we will further incorporate the analysis and processing of noise energy distribution in the frequency domain and refine the turbulence model to adapt to more complex turbulent environments.

In comparison with the standard instrument, a nearly linear deviation appeared in the positioning error. The primary causes were a minor height mismatch between the 6D system and the XL-80, and temperature variations resulting from different sensor locations. The maximum deviation in roll-angle measurement reached 4.6″, which can be attributed to two main factors: (1) the measured guideway exhibited a relatively large angular deviation, and the roll-sensitive waveplate might have introduced an additional phase shift when misaligned; this issue requires further detailed analysis; and (2) the nonuniform distribution of stray light on optical surfaces at different measurement distances could affect the energy-based roll-angle detection, resulting in deviation. The yaw and pitch angle measurements also exhibited slight linear deviations, which were likely caused by the detector being slightly out of the focal plane of the lens group. Therefore, further fine alignment and calibration of the detector installation are required to minimize this source of error.

## 5. Conclusions

This paper proposed a 6-DOF measurement method with air-turbulence compensation. To address the issue of straightness measurement degradation under long-distance conditions due to air turbulence, a dual-wavelength proportional cancellation model was developed. To suppress electronic noise, frequency-domain analysis and filtering were introduced into the data processing stage. The effectiveness of the proposed proportional cancellation model was verified through COMSOL numerical simulations. Furthermore, stability experiments demonstrated that the proposed compensation approach outperforms conventional SMA filtering methods, effectively mitigating the influence of air turbulence on measurement accuracy. Comparison experiments with a standard interferometric instrument confirmed the effectiveness of the proposed 6-DOF measurement system and its compensation method in terms of both repeatability and deviation consistency. The developed method can be widely applied to the geometric error measurement of long linear guideways and other precision positioning systems.

## Figures and Tables

**Table 1:** Summary of stability data.

	Horizontal Straightness/μm		Vertical Straightness/μm	
	Standard Deviation	Peak-to-Peak	Standard Deviation	Peak-to-Peak
Raw Data	1.2	12.9	1.5	17.9
SMA	0.9	8.1	1.2	10.9
Dual-Wavelength Compensation	0.4	3.4	0.4	4.4

**Table 2:** Summary of straightness comparison experiment results.

	Horizontal Straightness/μm			Vertical Straightness/μm		
	XL80	Moving Average Filtering	Dual-Wavelength Compensation	XL80	Moving Average Filtering	Dual-Wavelength Compensation
Maximum Deviation vs. XL-80	/	3.6	2.0	/	3.0	1.8
Repeatability	8.8	9.8	6.4	8.2	9.3	5.5

**Table 3:** Summary of other Degree-of-Freedom comparison experiments.

Result Statistics	Positioning/μm	Yaw/″	Pitch/″	Roll/″
6D Repeatability	6.4	1.7	2.1	4.3
Standard Instrument Repeatability	3.1	2.0	3.2	4.0
Maximum Deviation vs. Standard	2.5	4.1	1.7	4.6

## Data Availability

The original contributions presented in this study are included in the article. Further inquiries can be directed to the corresponding author.

## Appendix A

To validate the dual-wavelength proportional-cancellation method, we built a multiphysics model in COMSOL Multiphysics that couples fluid dynamics (CFD), heat transfer (HT) and geometrical optics (GO). The solver computes the velocity, temperature, density and refractive-index fields in a unified manner and traces rays through the refractive-index field to quantify beam wandering.

Computational domain and mesh: The computational domain is a closed rectangular cavity of 1.2 m × 0.6 m × 0.6 m. Rays propagate along the −Y direction. The fluid domain uses an unstructured tetrahedral mesh with inflation layers near the walls: maximum element size 0.10 m, minimum size 0.02 m, growth rate 1.5, and 12 wall layers with a normal growth factor of about 1.2. These settings ensure adequate resolution of the near-wall velocity and temperature gradients.

Fluid-dynamics model and boundary conditions: We adopt the Shear-Stress-Transport (SST) k-ω turbulence model, which blends k-ω near walls with k-ε in the free stream and resolves shear layers and recirculation regions well. Velocity inlet with a time-dependent profile: *u_x_* = 0.2(1 + 0.02sin(2*π* × 20*t*)), *u_y_* = 0.1sin(2*π* × 13*t*), *u_z_* = 0.1sin(2*π* × 17*t*). A small periodic disturbance is superposed in the streamwise direction to emulate unsteady inflow. The turbulence intensity is set to 1% (low-disturbance level) and the turbulence length scale to 0.01 m. Pressure outlet with gauge pressure 0 Pa, hydrostatic pressure compensation enabled and backflow suppression. All solid boundaries are treated as no-slip.

Heat transfer and thermal boundary conditions: The heat-transfer module is solved in steady state to generate a temperature gradient and the resulting density/refractive-index fields. A uniform heat flux *q_b_* = 2 W/m^2^ is applied on the bottom wall, the top wall is kept at T = 293.15 K, and the other walls are adiabatic. Thermal exchange at the walls produces a stable temperature gradient and buoyancy-driven natural convection, generating turbulence consistent with experimental conditions.

Refractive-index field: The refractive-index field is obtained from the density field using the Gladstone–Dale relation. The GO module reads the time-synchronized refractive-index field and performs ray tracing. A point source is located at the center of the inlet plane, emitting along the −Y direction. Ray bending due to ∇n is computed, and the spatial distribution of the beam spot on the outlet plane is used to evaluate turbulence-induced beam wander.
